# The Claims of Religion on Medical Men: A Discourse, Delivered in the Tenth Presbyterian Church, Philadelphia, on Sunday Evening, November 24, 1844

**Published:** 1845-01

**Authors:** 


*The Claims of Religion on Medical Men: A Discourse, delivered in the Tenth Presbyterian Church, Philadelphia, on Sunday evening, November 24th*, 1844. By B. A. BOARDMAN, Pastor of the Church. Philadelphia, 1844.

In a nation, the constitution and laws determine the genius of the government—the officers fix the character of its administration. If either be bad, the good which the other would exert, is countervailed; if *both* abound in imperfections, the condition of the governed is unfortunate; when both are excellent, the people are prosperous and happy. If we contemplate our profession in similitude with civil government, we find principles and practitioners, both of which are imperfect—both should be improved. Human nature has within itself many guaranties for the active cultivation of the former; and experience proves it not to be neglected. Love of gain and glory, curiosity and thirst for knowledge, have, and ever will stand responsible for a diligent attention to medicine as a science, or rather a group of sciences. But these principles of our nature, so far from pomoting the amelioration of character, which should prevail among those who practise the profession, too often arrest it. Thus avarice and ambition generate selfishness; and even philosophical curiosity, indulged as a passion, may draw the soul's blood to itself, and leave benevolence, generosity, and courtesy, to perish for want of that nutrition which their exercise only can supply. A full acquaintance with the principles of the profession, makes a man of science, but not a worthy physician. To become such, his moral sentiments must be developed not less than his intellectual faculties.

Unfortunately, our systems of education if not *limited* to the latter, direct their power chiefly upon it, and leave the former to grow up under whatever accidental influences may happily fall upon the student during the years of his pupilage and probation. In examinations for a degree, what medical institution ever thinks of extending its inquiries into the moral *character* of its candidates—how many even recognize, practically, the *reputation* of those whom they are about to usher into society, as its guardians of health and life? And, still, why should not his moral and social instincts, principles, and habits, be taken into account, when he asks admission into the ranks of a profession, the duties of which cannot be exercised acceptably to his brethren or society, if they are bad? It is expected, that before a young man enters a medical school, he should have made certain attainments in literature, not so much because he could not study science without them, as because their absence when he became a physician, would bring odium on the profession. But why should it not be equally, nay, still more, expected, that he should understand the principles of moral, social, and professional duty; and admit their obligation: his life up to that time having afforded evidence of their power?

But what are these principles? Where can they be found? We answer, unhesitatingly, in the BIBLE; which not only presents the whole, but offers them in connexion with all the sanctions of *both* moral philosophy and christianity. Does the former rest its obligations of duty on benevolence? The latter resolves every duty of man to man into that principle. Does it refer to a natural sentiment of justice? The latter every where commands the practice of that virtue. Does it rest upon utility? Christianity declares that Godliness is *profitable* for this life, not less than the life to come. In fact, whatever motives moral philosophy may have suggested for a life of rectitude, kind feeling, and gentle manners, the Bible recognizes the same; with the superadded obligation of a divine injunction. Thus it covers the whole ground of human duty, and recognizes every motive, which natural theology and moral philosophy admit, while it every where presents additional motives, which it declares to be revelations from God. In commending its study, we are not under the necessity of relying on the latter. Depending, at this moment, on the former alone, we may affirm, that the scriptures are the best book of moral philosophy, which the world has yet known; and that if every other were annihilated, nothing would be lost to the morality of mankind—no admissible motive left unrecognized, no personal duty unindicated, no maxim of social intercourse unsupplied. In what do all the disquisitions of moral philosophy terminate, but the principles and precepts of the Bible? What do they establish but the wisdom, merely human if you will, of a book far older than the most ancient among them? If it be a necessary part of education, to learn our duties towards those around us, and the *whole* are found scattered through the Bible, why should it not be studied? Why not derive our knowledge from a book whose antiquity, diversified authorship, solemnity of style, and consecrated name, are so well fitted to impress its precepts on the young heart?

It is a great error, that the study of the Bible is useless, unless it should eventuate in a conviction of its divine authorship. A faith in its wisdom, as a book of mere human origin, such a faith as its careful study will never fail to inspire, may exert on the character of the individual, a most genial influence. Under this view of its power, it is, that we recommend it to all who desire to cultivate their moral sentiments, their social feelings, and their manners. If *it* should not succeed, the case is hopeless. The deductions of moral philosophy will in vain essay to mould the heart, which has resisted the sublime and simple precepts of the Bible.

But there are higher and holier considerations to which we must now ascend. The Bible declares itself a book of revelation on the duty and destiny of man. If this be a fact, its profound and diligent study must be the duty of every human being. But here steps in scepticism, and pronounces that as it does not *know* that such was its origin, it feels no cbligation to study it. Now let us suppose, that when Dr. Jenner published to the world, that cow-pox was a preventive of small-pox, and would preserve the lives of the vaccinated, the profession at large had cherished the same scepticism, and refused to read his book, would they have been held guiltless? Would not the lives of the victims of small-pox have been required at their hands? Do we not carefully examine every new system of medicine, before we condemn it? Is it not according to the plainest dictates of common sense, that we should do so? What is the firm fabric of modern medicine, but the combined materials, which a critical examination of all the books which have been written for more than 2000 years, has extracted from so many masses of error, and perpetuated to the present time? Why then apply a different rule to the Bible? Why condemn it without examination? Manifestly because it professes to contain a revelation. Now we would ask the sceptic, whether he who made man, *could* not send him a revelation? Where is the warrant for denying that he might? Is it unreasonable? Reason certainly declares that *if* man were made, it would be a less exertion of power, to reveal to him certain maxims of duty towards his fellow man and his Creator. If a revelation, then, be possible, and the conclusion seems inevitable, it could not become known to the world, unless it received attention—were read and the evidences of its reality examined. But this is precisely what the majority of our profession have not done. Their infidelity is most unphilosophical, because they have concluded without examination: in violation of critical justice, for they have condemned without a hearing. If their disbelief should be correct, in the absolute, it is not *logically* correct, because not the result of a careful and candid investigation. To such an investigation we would call them. As scholars and philosophers, they should be ashamed of its omission; ashamed that they have concluded before they have collected and compared the testimony, absolutely necessary to a correct decision; before they have subjected all the facts, to the test of that logic on which they rely for the establishment of professional truth. When they have done this, should they not acquire a Christian faith, they will at least substitute a philosophical infidelity for the scepticism of ignorance. Into that cheerless region we should not have occasion to follow many of them, for its inhabitants are few, indeed, compared with those who wander in the benighted land of ignorance and doubt. We have seldom met with a single physician, who had earned citizenship in that frozen zone; while the number of the latter, although reduced from what it once was, is still sufficient to show, that multitudes repudiate the Bible, without having studied its doctrines or the evidences of its heavenly origin. It is to such, that we address this argument, and commend the discourses now before us.

That of Dr. Logan was delivered in a society composed of many popular, and some distinguished physicians of New Orleans. It is as creditable to their moral taste, that they should have ordered its publication, as it is to the fearless piety of the author, that he should have stood forth as the champion of the Bible, in a city where so many are *supposed* to neglect its study—so few to govern themselves by its divine precepts; where a code of honor is substituted for that of religion, and a *fighting* gentleman aspires to higher social rank than a *Christian* gentleman. That we may not be charged with exaggeration on this point, we present the testimony of our author. After announcing the subject of his address, he adds—

"I am especially urged to this theme at such a time and place, from the lamentable fact, that notwithstanding there are many practitioners in our country eminent for talents, illustrious for learning, and distinguished for skill, yet, I have reason to apprehend, too many are numbered among our ranks, who, by their reckless disregard and defiance of morals and religion, are ruining our influence, and bringing discredit upon the whole profession. Alas! how many are there, even in this city of chivalry and high-toned honor, who have no higher ideas of the dignity and responsibility of their vocation, than that it is a mere trade, a scuffle to gain popularity and business, and to pocket the dollars and cents!"

Our author proceeds to speak of the physical and spiritual nature of man, and justly insists that he who is ignorant of the latter, must necessarily be unqualified to combat many of the ills which beset the former. By a parity of reasoning, he would require in the priest a knowledge of the corporeal system. Indeed, in another part of his discourse, he intimates, that some advantages would attend the union of the two offices in the same individual; but in this suggestion we understand him to mean nothing more, than that medicine and theology have a natural and inseparable connection. On this point we shall quote his own just and impressive language:

"But let me be fairly understood on this point. Far, very far, am I from claiming for our science with ancient theosophy, a derivation from direct inspiration, nor with a sacrilegious ambition do I dare aspire to co-ordinate powers with the Christian evangelist. No. The period has rolled away into the dark distance of ages, when the then called 'divine art' of medicine was the appanage of a superstitious religion—when cooped up in catagories, it was doled out in eleemosynary morcels, by a corrupt priesthood, and when it was allied to the wildest metaphysics. No! Thank heaven! our art long since escaped from the dungeons of superstition—separated from the jargon of the schools—and now delivered from the chains of authority, by that true method of philosophising, which the genius of Bacon established; begins to feel the fecundating influence of the inductive logic. A just philosophy has shown, that man is no longer an isolated, but a related being; and in his dependencies and connections with other beings, and with the great system of nature, his affinities have been traced. And although he is the most complete and perfect type of organization, proceeding from the hand of the Creator, 'the very paragon of animals,' yet he must be studied by the same methods of investigation, as the other objects, even inanimate bodies of the universe. But there are other relations, also, in which the nature of man must be studied. He has a spiritual, as well as an animal existence. His frail and vulnerable body indicates his human nature, while his aspiring mind bears an immortal impress; and such is the wonderful sympathy that exists between these elements of being—this dual life, and so intimate is the vital connection, as we have before said, existing between them, that it is almost impossible to study the laws of the one, without becoming acquainted with the operations of the other. It is in this view of man's compound existence—this union of spirit and matter; the one, capable of immortality—of ever-living, ever blooming virtue; the other, heir of a life so brief, that its ephemeral period has been beautifully compared, by a fair writer,* to things most evanescent and mutable in nature: 'a vapor floating in the summer's sky—a little flower blooming in the morning, and in the evening withered and faded away—a dew-drop sparkling on a trembling leaf;'—it is in this view that the functions of the priest and the physician become blended—that their offices and duties become subsidiary to each other—that a oneness obtains between them. For, surely, if it falls to the province of our science to extend its salutary aid, 'to chase the ignorant fumes that mantle clearer reason,' it becomes the conscientious duty of the physician, not only to investigate all the mental phenomena and analyze their operations, but also to resort to the synthesis of all knowledge, psychical or physical, which may teach him to apply the proper therapeucy. And if he adopt the metaphysics and ethics of the Bible, which is universally acknowledged to be the simplest, clearest, and most luminous of all treatises on the philosophy, the government and destinies of man, how admirably would he approximate that sublimest ideal of human perfectibility—higher than which, human imagination never yet has soared—revealed in Him, who, while on earth, went about curing all manner of diseases, as well of mind as of body."

Our limits forbid additional extracts. In taking leave of our author, we cannot do less than ardently wish him success, in his efforts to awaken among his brethren a livelier sense of

---

* Margaret Mercer, authoress of Popular Lectures on Ethics, &c. Petersburg, 1841.

the importance of moral and religious cultivation. To his concluding sentence we give a heartfelt response. "Let us," says he, "in the vivid remembrance of our transitory existence, and in the practical application of those principles and precepts we have just been discussing, banish the rivalries of self love and of interest in controverted points, and never forget that we are in the presence of one who is the Arbiter of all truth—the Source of all light—the great Moral Physician of a disordered world!"

We turn from the commercial capital of the Southwest, to the medical metropolis of the East. Mr. Boardman's Sermon was delivered to the united classes of the University of Pennsylvania and the Jefferson Medical College, and published at their request. We are pleased to see at the head of each committee, the name of one of the most distinguished professors of its school.* We are also gratified at finding institutions, long and violently opposed to each other, at last concurring in a work of peace and piety. May they continue in this labor of love, and may their example be extensively imitated, until every school in the Union shall come to regard the religious instruction of its pupils as worthy of countenance. The most effective discipline of young men is the religious. Its fruits are industry, temperance, order, peace. It stands directly opposed to the prevalent vices of students, assembled in our cities from all parts of the country. It arrays itself against broils, duels, and the love of dissipated pleasures. It contributes to secure to every student, the full amount of his time and money, while it guaranties that tranquillity of mind, that exemption from the perturbations of appetite and passion, which is indispensable to calm and successful investigation.

But Mr. Boardman does not limit himself to transient benefits of this kind. He boldly affirms the duty of religious cultivation, not only for students but physicians—not merely for time but eternity. He reminds his hearers of their ruined

---

* Dr. N. Chapman and Dr. J. K. Mitchell.

moral condition, and calls upon them to drink at the only fountain of spiritual health and immortality—the *Bible.* He charges on them a neglect of that divine book, and refers, pointedly, to the prevalence of infidelity in the medical profession; which, however, he admits was greater in past times than at present. He then proceeds to enumerate the causes of this infidelity, and wishing to present our readers with a specimen of his sermon, we shall extract the portion which contains his views on this branch of his discourse:

"It has, however, been admitted, that there are facts in the earlier annals of the science, which *seem* to countenance the charge under consideration, that medical studies involve a lurking tendency to infidelity. I use the expression 'seem,' because in so far as the science itself is concerned, the imputation *must* be groundless. The principles of every science were established by the Divine Author of Christianity, and cannot, therefore, conflict with any of its doctrines or requirements. The legitimate tendency of all scientific investigations, is to lead the mind up to the Great First Cause, and to predispose it to bow to His mandates, whenever and howsoever they may be communicated. But that these studies are often perverted from their appropriate end, no one can deny. And this has perhaps happened as frequently in medicine as in any other science. Various reasons have been assigned for this fact.

"1. There is the absorbing nature of the demands the profession makes upon the time and attention of its votaries, who are thereby deprived (as they suppose) of the opportunity for examining the subject of religion, and indeed rendered averse to it.

"2. Successful scientific researches are apt, unless regulated by religious considerations, to inspire men with inflated views of the sufficiency of human reason on *all* subjects; and thus, from questioning the necessity, they may easily come to deny the fact of a Divine revelation.

"3. The habit of reasoning from induction and analogy which belongs to every scientific physician, may unfit them, in a measure, for examining with impartiality the argument from miracles which constitutes so material a portion of the external evidences of Christianity.*

---

* See Gisborne on Men, vol. ii., 194.

"4. I say it with regret, but nothing has impressed my own mind more unpleasantly, in the little attention I have given to medical works, than the want of a distinct recognition of the Creator's power and agency, on occasions when it would not only be natural for the writer to refer to the Deity, but even when the idea was evidently in his own mind, and could not be suppressed without an effort. 'The student of medicine,' says an ingenious writer, 'is often called on to bring his gift and deposit it, like the Athenian, on the altar of an 'unknown god.' A cloudy image, entitled 'nature,' is raised in the mind, to which high attributes of power, wisdom, and goodness, are often ascribed.'* He might have added that the tendency of this habit, if persisted in, must be, in minds peculiarly constituted, to create a vague impression which may ultimately grow into a conviction, that this obscure divinity, 'nature,' is really the only Deity.

"5. Physicians are conversant with those scenes of suffering which, above any others, appeal to our sympathies. These scenes do not necessarily blunt their humane feelings, but they can hardly fail of producing a decisive effect, for good or evil, upon their moral sensibilities. It may be worthy of consideration, whether familiarity with such spectacles has not sometimes assisted in fortifying them against the requisitions of Christianity, and even hastening them into infidelity.

"6. The exposure of young men, while in training for the profession, to the temptations of large cities, and the consequent formation of dissolute habits, has, no doubt, been a hotbed of skepticism.

"7. The neglect of divine worship on the Sabbath, and of the other means of grace, has, it is to be feared, contributed in no small degree to foster infidelity among physicians. I shall enter into no argument on this point. It will probably be conceded, on all hands, that physicians frequently absent themselves from the sanctuay when no call of necessity or mercy pleads for it; that the occasional neglect of the house of God easily glides into a habit; and that this habit tends, by a natural process, to impair all suitable sense of religion, and to generate infidelity.

"Such are some of the grounds on which the prevalence of skepticism among medical men has been explained. They show that however guiltless the science itself may be of the

---

* "Is Medical Science favorable to Skepticism?" An essay, by James W. Dale, M. D.

infidel tendency ascribed to it, there is real danger in the path of the physician. They certainly furnish a strong argument in favor of an early and persevering attention to the claims of religion, on the part of the profession. This would not only secure them from the ruinous illusions of infidelity, but furnish a triumphant vindication of their art from the stigma which has been unjustly affixed to it."

While we concur with our reverend author in these causes, we are constrained to express the belief, that ignorance of the Bible is a greater cause of skepticism than the whole; for its diligent study would, in fact, obviate several of them; and without such study, a faith in its divine authorship is not likely to be established, in minds accustomed to read and think.

The remainder of his interesting sermon is composed chiefly of an inquiry into the irritations, anxieties and troubles of medical men, which religion would either palliate or remove; into the increased usefulness to the world, of him who adds piety to science; and of solemn exhortations to personal religion, as a preparation for death. May they animate all who heard them, "to do justice, love mercy, and walk humbly with God."

In conclusion, we cannot refrain from asking—what would be the character of the profession, if all its members lived under this divine law of justice and mercy to all around them—of reverence and humility before their Heavenly Father? Bright and beautiful, indeed, would it then appear to all the world! Beloved, honored, gratefully remembered by man—approved by God himself. Cold-blooded derelictions of duty to the sick would then be replaced by early, anxious, and faithful attention—candor would supplant concealment and deception—courtesy and kind words, warm from the heart, would solace, sustain, and cheer the afflicted. Physicians would then confide in each other, where they now watch with jealousy. Unavoidable collisions would be amicably adjusted, and each would act towards every other as he would be dealt with by them. The medical man would not, then, heartlessly withdraw when the man of God was about to offer up the prayer of faith and hope, over the expiring sinner; but, uniting in the last sad and solemn duty which humanity can perform, would, by his very presence, mitigate the pangs which his skill could not avert, and fill the soul of the dying man with emotions of gratitude.

